# Culprit Plaque Characteristics in Patients With Sleep‐Disordered Breathing Undergoing Percutaneous Coronary Intervention: An Intravascular Ultrasound Study

**DOI:** 10.1161/JAHA.118.009826

**Published:** 2018-09-29

**Authors:** Hideki Wada, Tomotaka Dohi, Takatoshi Kasai, Shoichiro Yatsu, Ryo Naito, Yoshiteru Kato, Iwao Okai, Hiroshi Iwata, Kikuo Isoda, Shinya Okazaki, Katsumi Miyauchi, Hiroyuki Daida

**Affiliations:** ^1^ Department of Cardiovascular Medicine Juntendo University Graduate School of Medicine Tokyo Japan

**Keywords:** attenuation plaque, coronary artery disease, intravascular ultrasound, percutaneous coronary intervention, sleep‐disordered breathing, Coronary Artery Disease, Percutaneous Coronary Intervention, Imaging

## Abstract

**Background:**

Sleep‐disordered breathing (SDB) is a novel cardiovascular risk factor. However, the coronary plaque characteristics of patients with SDB with coronary artery disease are still unclear.

**Methods and Results:**

This study included 289 consecutive patients with coronary artery disease undergoing percutaneous coronary intervention. Plaque characteristics of the culprit lesion were assessed by preintervention intravascular ultrasound. The presence of SDB was defined as a 3% oxygen desaturation index of ≥15 events per hour measured by nocturnal pulse oximetry. Of 289 patients, the median 3% oxygen desaturation index was 9.6 (interquartile range, 5.1–16.6), and 88 patients (30.4%) were defined as having SDB. Compared with the no‐SDB group, the SDB group had a larger total atheroma volume of the culprit lesion (224.5 mm^3^ versus 190.8 mm^3^, *P*=0.05). The median maximum attenuation and calcification angle were 140° and 130°, respectively. Attenuated plaque with a maximum attenuation angle >140° was more frequently observed in the SDB group compared with the no‐SDB group (34.9% versus 22.6%; *P*=0.03). However, there were no statistically significant differences between groups in the maximum calcium angle and the frequency of calcific plaques with a maximum calcium angle >130°. Multivariable logistic regression analysis showed that the presence of SDB was a significant predictor of a greater ultrasound attenuation angle (>140°) (odds ratio, 1.86; 95% confidence interval, 1.02–3.39; *P*=0.04).

**Conclusions:**

SDB was associated with larger atheroma plaque volume and a greater ultrasound attenuation, which are discriminators of plaque vulnerability. Further studies are needed to clarify the effects of SDB treatment on coronary plaque lesions.

**Clinical Perspective**

**What Is New?**

- Patients with sleep‐disordered breathing (SDB) had larger total atheroma volume of the culprit lesion than patients without SDB.
- Attenuated plaque with a maximum attenuation angle >140° (the median maximum attenuation angle) was more frequently observed among patients with SDB compared with patients without SDB.
- The presence of SDB was an independent predictor of a greater ultrasound attenuation angle.

**What Are the Clinical Implications?**

- The results of the present study could provide a potential mechanism for the association between the presence of SDB and poor clinical outcomes among patients with coronary artery disease.
- The mechanical intervention in patients with SDB may reduce coronary atheroma volume or the degree of ultrasound attenuation.

Sleep‐disordered breathing (SDB) is a severe chronic condition in which partial or complete cessation of breathing occurs many times throughout the night. The prevalence of SDB associated with accompanying daytime sleepiness is ≈3% to 7% for adult men and 2% to 5% for adult women in the general population.^1^ By contrast, in patients with cardiovascular disease, its prevalence has been reported to be 30% to 57%.^2^, ^3^, ^4^ SDB has been recognized as a strong risk factor for the development of hypertension, heart failure, arrhythmias, and stroke, especially in men.^5^, ^6^, ^7^ Recently, some observational studies reported that obstructive sleep apnea was associated with adverse cardiac events in patients with coronary artery disease (CAD) who underwent percutaneous coronary intervention (PCI).^8^, ^9^ Therefore, the presence of SDB is now considered an independent cardiovascular risk factor.^10^ However, the mechanisms of the relation between CAD and SDB are unclear. Patients with SDB tend to have hypertension or heart failure, which may affect clinical outcomes in this population. SDB has also been reported to be associated with coronary atherosclerosis.^11^, ^12^ A previous study demonstrated an association between SDB and larger total atheroma volume in the target coronary artery but not the incidence of thin‐cap fibroatheroma.^12^ However, coronary plaque tissue composition may not be well characterized based only on the atheroma volume of the lesion. We therefore sought to evaluate the association between SDB and culprit coronary plaque findings, including quantitative and qualitative analysis by gray‐scale intravascular ultrasound (IVUS) in patients with CAD who underwent PCI.

## Methods

### Study Population

**Figure 1:**
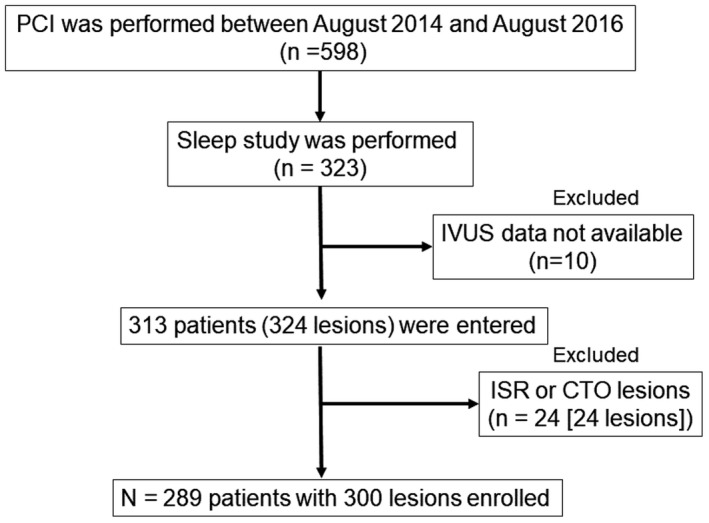
Flow chart of the study. A sleep study was performed in 323 patients between August 2014 and August 2016. A total of 289 patients (300 lesions) were enrolled. CTO indicates chronic total occlusion; ISR, in‐stent restenosis; IVUS, intravascular ultrasound; PCI, percutaneous coronary intervention.

In this prospective, single‐center, observational study, 289 consecutive patients with CAD who were admitted to Juntendo University Hospital were enrolled from August 2014 to August 2016 (Figure 1). The inclusion criteria were as follows: (1) patients who underwent PCI under IVUS guidance to treat a de novo culprit lesion and (2) patients who were evaluated for SDB by overnight pulse oximetry during hospitalization. The exclusion criteria were as follows: (1) patients who had an in‐stent restenosis or a chronic total occlusion lesion; (2) patients who were already diagnosed with SDB or treated by continuous positive airway pressure (CPAP) therapy; and (3) patients in whom adequate IVUS images or data from a sleep study examination were not obtained.

Demographic data, coronary risk factors, and medication use were collected from our institutional database. Blood samples were collected in the early morning after overnight fasting, and blood pressure was measured on admission. Patients with blood pressure >140/90 mm Hg or those receiving antihypertensive drugs were regarded as hypertensive. Dyslipidemia was defined as low‐density lipoprotein cholesterol ≥140 mg/dL, high‐density lipoprotein cholesterol ≤40 mg/dL, triglycerides ≥150 mg/dL, or current treatment with statins and/or lipid‐lowering agents.^13^ Diabetes mellitus was defined as either hemoglobin A_1c_ ≥6.5% or medication with insulin or oral hypoglycemic drugs. Chronic kidney disease was defined as an estimated glomerular filtration rate <60 mL/min per 1.73 m^2^ as calculated using the Modification of Diet in Renal Disease equation modified with a Japanese coefficient using baseline serum creatinine.^14^ A current smoker was defined as a person who was a smoker at the time of PCI or who had quit smoking within 1 year before PCI. Acute coronary syndrome (ACS) was defined as an acute myocardial infarction and unstable angina. Acute myocardial infarction was characterized by elevated cardiac enzymes. Unstable angina was diagnosed in the presence of ischemic symptoms without release of enzymes and biomarkers associated with myocardial necrosis.

This study was approved by the Juntendo University Ethnic Committee and was performed in accordance with the Declaration of Helsinki. All participants provided written informed consent. The data, analytic methods, and study materials will not be made available to other researchers for purposes of reproducing the results.

### IVUS Image Acquisition and Analysis

In all study patients, the culprit plaque lesion was imaged by preintervention IVUS. A culprit lesion segment was defined as the lesion that was stented by comparing pre‐ and post‐PCI IVUS. Two commercially available IVUS systems and catheters were used in this study: a mechanical rotating 40‐MHz transducer (Atlantis Pro2, Boston Scientific Corporation, Natick, MA, USA, and View It, Terumo Corporation, Tokyo, Japan). After intracoronary administration of 0.1 to 0.2 mg nitroglycerin, IVUS image acquisition was performed in the culprit lesion segment before balloon dilatation or after small (1.5–2.0 mm) balloon dilatation. All IVUS Ppullbacks were performed automatically at 0.5 mm/s.

All measurements were fulfilled at the end of this study. Quantitative and qualitative IVUS analyses were performed according to the criteria of American College of Cardiology Clinical Expert Consensus Document on Standards for Acquisition, Measurement and Reporting of Intravascular Ultrasound Studies,^15^ and morphological features were diagnosed by careful review of IVUS images and the agreement of the 2 independent experienced cardiologists (H.W. and T.D.) who were blinded to clinical data, including sleep study results. Offline analyses of all imaged segments were performed using computerized planimetry software (QIVUS; Medis Medical Imaging System, Leiden, the Netherlands).

**Figure 2:**
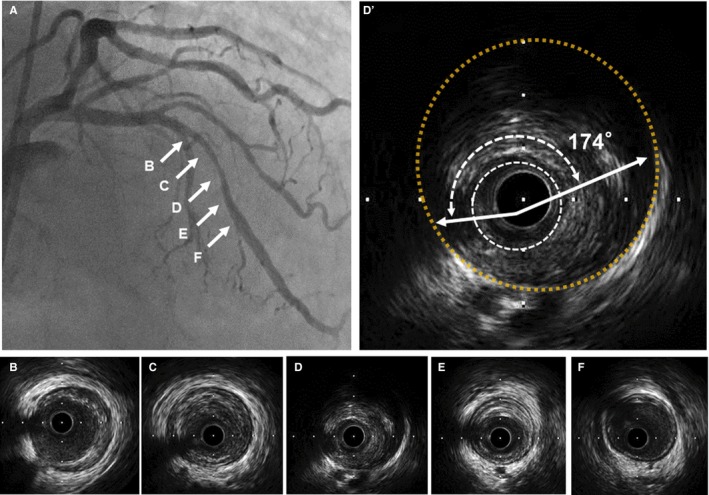
Representative case of intravascular ultrasound‐detected attenuated plaque. Findings of an 81‐year‐old male with stable coronary artery disease and sleep‐disordered breathing are shown. On coronary angiography, severe coronary stenosis was observed (A), and on intravascular ultrasound examination (B through F), positive remodeling plaque (remodeling index, 1.49) with ultrasound attenuation (D’, maximum ultrasound attenuation angle, 174°) was observed in the mid left descending artery. In this case, 3% oxygen desaturation index measured by nocturnal pulse oximetry was 53.2 events per hour of sleep.

Quantitative IVUS measurements included the external elastic membrane (EEM), lumen cross‐sectional area (CSA), and the plaque plus media (EEM‐lumen) CSA. The plaque burden was calculated as the plaque plus media CSA divided by the lesion EEM CSA multiplied by 100. Total atheroma volume (TAV) was calculated as the sum of the differences between the EEM and lumen areas across all segments analyzed. Percent atheroma volume was calculated as the proportion of the entire lesion segment occupied by the atherosclerotic plaque. Qualitative IVUS analysis included plaque rupture (presence of a cavity that communicated with the lumen with an overlying residual fibrous cap), thrombus (an intraluminal mass, often with a layered, lobulated, or pedunculated appearance), calcification (brighter plaque than adventitia with acoustic shadowing), and ultrasound attenuation behind the plaque in the absence of calcification. The maximum angle of the ultrasound attenuation and calcification of the lesion was also measured. The remodeling index was calculated as the EEM CSA at the minimal lumen area site divided by the average of the proximal and distal reference EEM CSA. A representative case is shown in Figure 2.

### Overnight Sleep Study

Nocturnal pulse oximetry was performed for 1 night in the hospital using finger pulse oximetry (Pulsox Me‐300; Teijin Pharma, Tokyo, Japan). Arterial oxyhemoglobin saturation was recorded using a finger probe at a 1‐Hz sampling frequency and a 5‐second averaging time. We scored these recordings using specific software (DS‐M; Teijin Pharma, Tokyo, Japan), with manual corrections performed by a trained scorer who was blinded to the baseline subject information. Mean and minimum arterial oxyhemoglobin saturation, the time spent with arterial oxyhemoglobin saturation below 90%, and the number of desaturations per hour, which were expressed as the oxygen desaturation index (ODI), were computed separately. The 3%ODI was selected as an index of oxygen desaturation, representing the number of events per hour of the recording time in which the patient's blood oxygen level fell by ≥3%. In this study, we defined significant SDB as having a 3%ODI of ≥15 events per hour.^16^ Patients were divided into 2 groups: the no‐SDB group and the SDB group.

### Statistical Analysis

Differences in demographic characteristics were compared between the 2 groups (3%ODI <15 [no‐SDB group] and ≥15 [SDB group]). Quantitative data are presented as means±standard deviation or medians (interquartile range). Categorical variables are presented as frequencies. Continuous variables were compared using an unpaired *t* test or the Mann‐Whitney *U* test. Categorical variables (presented as frequencies) were compared using the chi‐square test. Multivariable logistic regression analysis controlling for potential confounders (age, sex, hypertension, diabetes mellitus, dyslipidemia, smoking status, body mass index, chronic kidney disease, and ACS on admission) was used to calculate the odds ratio (OR) of the attenuated plaque (maximum attenuated angle ≥140°) between 2 groups (3%ODI <15 and ≥15). Differences were considered significant at *P*<0.05. Statistical analyses were carried out using JMP version 12.0 software (SAS Institute, Cary, NC, USA).

## Results

### Baseline Patient Clinical Characteristics

In the 289 patients, the median and mean 3%ODI were 9.6 (interquartile range, 5.1, 16.6) and 12.3±10.1, respectively. Of these, 88 patients (30.4%) were defined as having SDB (3%ODI ≥15). Baseline demographics are summarized in Table 1. The mean age of patients was 67.4 years, 83.7% were men, and 15.9% presented with ACS. Patients with SDB were more likely to be male and to have a higher body mass index, higher triglycerides and fasting blood glucose levels, and lower high‐density lipoprotein cholesterol levels. There were no differences in the prevalence of hypertension, diabetes mellitus, or chronic kidney disease between the SDB group and no‐SDB group.

**Table 1:** Mean Baseline Characteristics of Patients (N=289)

	Overall (n=289)	3%ODI <15 (n=201)	3%ODI ≥15 (n=88)	*P* Value
Age, y	67.4±11.3	66.7±11.3	69.1±11.3	0.09
Male (%)	242 (83.7)	161 (80.1)	81 (92.1)	0.01
Body mass index, kg/m^2^	24.8±4.0	24.2±3.6	26.1±4.6	0.0002
Hypertension (%)	219 (75.8)	147 (73.1)	72 (81.8)	0.11
Diabetes mellitus (%)	114 (39.6)	75 (37.3)	39 (44.8)	0.23
Dyslipidemia (%)	223 (77.2)	150 (74.6)	73 (83.0)	0.12
Current smoker (%)	66 (22.8)	46 (22.9)	20 (22.7)	0.98
Family history of coronary artery disease (%)	73 (25.4)	47 (23.5)	26 (29.9)	0.25
Acute coronary syndrome presentation	46 (15.9)	32 (15.9)	14 (15.9)	0.998
Chronic kidney disease, %	72 (24.9)	49 (24.4)	23 (26.1)	0.75
Prior statin use, %	215 (74.4)	148 (73.6)	67 (76.1)	0.65
Low‐density lipoprotein cholesterol, mg/dL	94.7±31.3	96.1±28.1	91.4±37.7	0.24
High‐density lipoprotein cholesterol, mg/dL	44.9±12.3	45.9±12.8	42.7±10.8	0.04
Triglycerides, mg/dL	130.4±66.9	124.3±58.8	144.4±81.1	0.02
Hemoglobin A_1c_, %	6.2±0.8	6.2±0.8	6.4±0.7	0.07
Fasting blood glucose, mg/dL	103.8±25.8	101.0±22.2	110.3±31.8	0.005
High‐sensitivity C‐reactive protein, mg/dL^a^	0.07 [0.03, 0.21]	0.06 [0.03, 0.18]	0.08 [0.04, 0.24]	0.08
Estimated glomerular filtration rate, mL/min per 1.73 m^2^	72.5±25.4	73.3±24.9	70.4±26.5	0.38
Sleep parameters				
3%ODI, events/h^a^	9.6 [5.1, 16.6]	6.6 [4.1, 10.1]	21.4 [17.9, 28.1]	<0.0001
4%ODI, events/h^a^	6.0 [2.9, 11.8]	3.8 [2.3, 6.3]	15.4 [12.4, 20.3]	<0.0001
Mean SpO_2_	94.3±1.7	94.7±1.5	93.3±1.8	<0.0001
Time of SpO_2_ <90%, %^a^	1.0 [0.2, 4.5]	0.4 [0.1, 1.7]	5.3 [2.0, 15.1]	<0.0001

ODI indicates oxygen desaturation index; and SpO_2_, arterial oxygen saturation.

a Median [interquartile range].

### Quantitative and Qualitative IVUS Findings

A total of 300 culprit lesions were analyzed. Findings of lesion‐level coronary angiography and IVUS are shown in Table 2. Overall, median minimal lumen area, percent atheroma volume, and remodeling index were 2.1 mm^2^, 62.5%, and 1.1, respectively. There were no significant differences in quantitative parameters, such as plaque burden at minimal lumen area, percent atheroma volume, and remodeling index between the SDB group and the no‐SDB group. However, patients in the SDB group tended to have a larger total atheroma volume of the culprit lesion than patients in the no‐SDB group (224.5 mm^3^ versus 190.8 mm^3^; *P*=0.05). There was no significant correlation between 3%ODI and percent atheroma volume (*r*=0.08; *P*=0.14). In addition, no relationship between 3%ODI and remodeling index was observed (*r*=−0.07; *P*=0.24).

**Table 2:** Mean Coronary Angiography and IVUS Findings of Lesions

	Overall (n=300)	3%ODI <15 (n=208)	3%ODI ≥15 (n=92)	*P* Value
Quantitative coronary angiography				
Culprit vessel				0.10
Left anterior descending artery (%)	138 (46.0)	95 (45.7)	43 (46.7)	
Right coronary artery (%)	93 (31.0)	57 (27.4)	36 (39.1)	
Left circumflex artery (%)	59 (19.7)	48 (23.1)	11 (12.0)	
Other	10 (3.3)	8 (3.8)	2 (2.2)	
Total length of lesions, mm^a^	23.9 [16.1, 33.1]	23.1 [16.0, 33.0]	24.4 [18.1, 36.4]	0.13
IVUS findings				
Quantitative parameters				
MLA, mm^2^ a	2.1 [1.8, 2.6]	2.1 [1.8, 2.7]	2.1 [1.7, 2.5]	0.46
EEM at MLA site, mm^2^ a	12.7 [9.5, 16.4]	12.6 [9.2, 16.4]	13.0 [9.6, 16.4]	0.80
Area stenosis at MLA, %^a^	81.9 [75.9, 87.0]	81.3 [75.1, 86.8]	82.9 [76.9, 87.7]	0.23
Percent atheroma volume, %^a^	62.5 [54.5, 68.2]	62.4 [54.3, 67.5]	62.9 [55.5, 68.8]	0.24
Total atheroma volume, mm^3^ a	198.9 [138.8, 300.2]	190.8 [132.2, 290.0]	224.5 [147.3, 308.5]	0.05
Total atheroma volume_normalized_, mm^3^ a	206.7 [150.1, 258.0]	201.8 [147.3, 261.4]	215.3 [158.3, 250.7]	0.36
Remodeling index^a^	1.1 [1.0, 1.2]	1.1 [1.0, 1.2]	1.1 [1.0, 1.2]	0.12
Qualitative assessment and parameters				
Culprit plaque type				0.92
Soft	92 (30.7)	63 (30.3)	29 (31.5)	
Fibrous	84 (28.0)	60 (28.9)	24 (26.1)	
Calcific	71 (23.7)	50 (24.0)	21 (22.8)	
Mixed	53 (17.7)	35 (16.8)	18 (19.6)	
Plaque rupture (%)	66 (22.0)	45 (21.6)	21 (22.8)	0.82
Thrombus on IVUS (%)	34 (11.3)	23 (11.1)	11 (12.0)	0.82
Calcified nodule (%)	53 (17.7)	33 (15.9)	20 (21.7)	0.22
Ultrasound attenuation (%)	162 (54.0)	111 (53.4)	51 (55.4)	0.74
Maximum attenuation angle, °^a^	138 [107, 175]	129 [103, 167]	148 [114, 198]	0.06
Maximum attenuation angle ≥ median 140°	79 (34.8)	47 (22.6)	32 (34.9)	0.03
Maximum calcium angle, °^a^	130 [84, 242]	137 [85, 262]	124 [81, 224]	0.47
Maximum calcium angle ≥ median 130°^a^	199 (66.3)	75 (36.1)	26 (28.3)	0.19

EEM indicates external elastic membrane; IVUS, intravascular ultrasound; MLA, minimal lumen area; and ODI, oxygen desaturation index.

a Median [interquartile range].

In the overall qualitative analysis, 30.7% and 23.7% of the plaques were described as soft and calcific plaques, respectively. There were no significant differences in the culprit plaque type between groups. Similarly, the frequency of plaque rupture and calcified nodules did not differ between groups. The frequency of ultrasound attenuation was similar between groups (55.4% versus 53.4%; *P*=0.74), and the maximum attenuation angle tended to be greater in the SDB group than the no‐SDB group (148° versus 129°, *P*=0.06). The median maximum attenuation and calcification angle were 140° and 130°, respectively. Attenuated plaque with a maximum attenuation angle >140° was more frequently observed in the SDB group compared with the no‐SDB group (34.9% versus 22.6%; *P*=0.03). On the other hand, there were no statistically significant differences between groups in the maximum calcium angle (124° versus 137°; *P*=0.47) and the frequency of calcific plaques with a maximum calcium angle >130° (28.3% versus 36.1%; *P*=0.19). Interestingly, among plaques in patients with ACS (n=46 lesions, 15.3%), the frequency of a maximum attenuation angle >140° in the SDB group was significantly higher than the no‐SDB group (71.4% versus 28.6%; *P*=0.01).

### Multivariable Logistic Regression Model

The univariate logistic regression analysis showed that the presence of SDB was significantly associated with a greater ultrasound attenuation angle (>140°) (OR, 1.83; 95% confidence interval, 1.06–3.13; *P*=0.03). In the multivariable model (Table 3), independent predictors of a greater ultrasound attenuation angle were the presence of SDB (adjusted OR, 1.86; 95% confidence interval, 1.02–3.39; *P*=0.04), ACS presentation (OR, 2.54; 95% confidence interval, 1.26–5.11; *P*=0.01), and chronic kidney disease (OR, 0.40; 95% confidence interval, 0.18–0.84; *P*=0.01).

**Table 3:** Multivariate Logistic Regression Analysis for a Median Attenuation Angle ≥140°

	Odds Ratio	95% CI	*P* Value
3%ODI ≥15	1.86	1.02–3.39	0.04
Acute coronary syndrome on admission	2.54	1.26–5.11	0.01
Chronic kidney disease	0.40	0.18–0.84	0.01
Male	2.17	0.88–6.15	0.09
Dyslipidemia	0.64	0.33–1.25	0.19
Diabetes mellitus	1.41	0.79–2.50	0.24
Current smoker	1.45	0.75–2.77	0.27
Body mass index	0.97	0.89–1.05	0.45
Age	1.01	0.98–1.04	0.53
Hypertension	1.22	0.64–2.42	0.55

CI indicates confidence interval; and ODI, oxygen desaturation index.

## Discussion

The major findings of this study were as follows: (1) Patients in the SDB group had larger total atheroma volume of the culprit lesion than patients in the no‐SDB group; (2) attenuated plaque with a maximum attenuation angle >140° was more frequently observed in the SDB group compared with the no‐SDB group; and (3) multivariable logistic regression analysis showed that the presence of SDB was an independent predictor of a greater ultrasound attenuation angle. Overall, our results showed that SDB was associated with larger plaque volume and a greater ultrasound attenuation, which are discriminators of plaque vulnerability.

SDB has been associated with coronary artery atherosclerosis. Several studies using computed tomography imaging have shown a positive association between SDB and coronary artery calcium, noncalcified coronary plaque, or coronary artery plaque volume.^11^, ^17^, ^18^ Furthermore, previous studies demonstrated a relation between coronary plaque volume evaluated by IVUS imaging and the presence of SDB.^12^, ^19^ The correlation between atheroma plaque volume and SDB was also shown in our study. Several possible mechanisms linking sleep apnea to atherosclerosis have been proposed. Intermittent hypoxia and reoxygenation can induce sustained sympathetic activation, increased oxidative stress, activation of inflammatory mediators, or endothelial dysfunction.^20^ Indeed, in the present study, high‐sensitivity C‐reactive protein levels of patients with SDB patients were likely to be higher compared with patients without SDB.

Although the relation between SDB and coronary atherosclerosis has been reported before, it is uncertain whether SDB affects coronary plaque vulnerability. To the best of our knowledge, the present investigation is the first to find an association between SDB and the degree of ultrasound attenuation. Echo‐attenuated plaque is frequently observed at culprit lesions in patients with ACS and is associated with deterioration of coronary flow and periprocedural myocardial infarction following PCI.^21^, ^22^, ^23^ Pu et al compared IVUS signatures with pathological histology in a large series of postmortem human coronary samples and showed that IVUS attenuation was indicative of either a fibroatheroma containing a large necrotic core or pathological intimal thickening with a large lipid pool.^24^ They also demonstrated that the angle of IVUS attenuation was significantly correlated with the angle of the histopathologic necrotic core or lipid pool. The size of the lipid pool or necrotic core has been significantly associated with plaque rupture.^25^ Therefore, the results of the present study could provide a potential mechanism for the association between the presence of SDB and poor clinical outcomes among patients with CAD.

CPAP, the standard treatment for SDB, has been associated with decreased cardiovascular events, alleviated daytime sleepiness, and improved quality of life.^26^ In a randomized trial, 4 months of treatment with CPAP significantly reduced both carotid intima‐media thickness and arterial stiffness, which was associated with improving validated markers of atherosclerosis.^27^ In an observational study, Cassar et al reported that patients with treated SDB who underwent PCI had a significantly lower cardiac mortality than did patients with untreated SDB.^28^ However, recent randomized trials could not demonstrate that CPAP therapy reduced cardiovascular events in patients with SDB and cardiovascular diseases.^29^, ^30^ Ongoing clinical trials such as the ISAACC (Continuous Positive Airway Pressure [CPAP] in Patients With Acute Coronary Syndrome and Obstructive Sleep Apnea [OSA]) study (http://ClinicalTrials.gov number, NCT01335087) will shed further light on the effect of CPAP in patients with obstructive sleep apnea and ACS. From the results of the present study, this mechanical intervention in patients with SDB may reduce coronary atheroma volume or the degree of ultrasound attenuation.

This study had several limitations. First, SDB was assessed using a finger pulse oximetry instead of polysomnography. Although polysomnography is the gold standard in the diagnosis of SDB, the accessibility of polysomnography is relatively limited, and it is difficult to use in daily clinical practice. Second, we combined patients with stable CAD and ACS in the present study. ACS is generally associated with attenuated plaque and larger atheroma volume. Therefore, we added ACS on admission into the multivariate model in addition to other risk factors.

## Conclusions

This study found that SDB was associated with larger atheroma plaque volume and a greater ultrasound attenuation, which are discriminators of plaque vulnerability. Further studies are necessary to clarify the effects of SDB treatment on coronary plaque lesions.

## Disclosures

None.
